# Levees for a hundred-year flood: impact of a syndrome-based antimicrobial stewardship intervention for coronavirus disease 2019 on antimicrobial use and resistance

**DOI:** 10.1017/ash.2024.383

**Published:** 2024-09-18

**Authors:** Alfredo J. Mena Lora, Rodrigo Burgos, Dylan Huber, Lawrence Sanchez, Mirza Ali, Candice Krill, Eden Takhsh, Susan C. Bleasdale

**Affiliations:** 1 University of Illinois at Chicago, Chicago, IL, USA; 2 Saint Anthony Hospital, Chicago, IL, USA

## Abstract

Coronavirus disease 2019 can be indistinguishable from lower respiratory tract infections (LRTIs) caused by other viral and bacterial agents. This likely contributed to antimicrobial use (AU) and antimicrobial resistance (AMR) during the pandemic. Our antimicrobial stewardship program targeted the selection and duration of therapy for LRTIs and led to a reduction in AU and AMR.

## Background

Lower respiratory tract infections (LRTIs) caused by severe acute respiratory coronavirus virus 2 presented a unique challenge to healthcare systems worldwide.^1,2^ Coronavirus disease 2019 (COVID-19) symptoms can be indistinguishable from lower respiratory tract infections (LRTIs) caused by other viral or bacterial agents.^1^ This posed a diagnostic challenge during the initial stages of the pandemic when data on coinfections was lacking.^3^ Consequently, clinicians faced difficulties in accurately distinguishing COVID-19 from other etiologies of LRTIs, leading to a surge in antimicrobial use (AU).^4^ This antimicrobial pressure may have contributed to a significant increase in antimicrobial resistance (AMR) during the pandemic, highlighting the importance of finding effective antimicrobial stewardship (ASP) strategies for respiratory virus pandemic preparedness and response.^5^

ASP can optimize AU, enhance patient outcomes, and curb the development of AMR.^6^ We incorporated COVID-19 elements into our syndrome-based ASP in an urban safety-net community hospital targeting selection and duration of therapy. Our study aims to understand the impact of this program on AU and broad-spectrum antipseudomonal beta-lactams (APBL) use during the COVID-19 pandemic.

## Methods

### Study design and setting

We conducted a single-center retrospective review of AU data at a 151-bed safety-net community hospital located on the west side of Chicago that provides medical, surgical, pediatric, and obstetrics-gynecology inpatient services. Our facility has 1 ID physician, 1 lead ASP pharmacist, and 4 full-time pharmacists without ID postgraduate training.

### Intervention

We incorporated COVID-19-specific elements into a previously established syndrome-based ASP.^7^ To achieve this, guidelines on antimicrobial selection and duration for patients admitted with known or suspected COVID-19 pneumonia were developed, and educational materials for physicians and pharmacists were disseminated. Guidelines were shared via hospital intranet, paper, and posters in clinical areas. COVID-19 electronic medical record order sets were developed (Supplement 1). The order sets discouraged the routine use of antimicrobials for COVID-19, advised against broad-spectrum APBL use, and recommended limited durations of therapy. The prospective audit and feedback (PAF) component of our intervention targeted LRTIs and COVID-19 therapies, providing real-time feedback and recommendations to healthcare providers. As the literature evolved and clarified the low risk of bacterial coinfection with COVID-19 on presentation, PAF strongly discouraged unnecessary AU on admission after July 1, 2020, and all subsequent COVID-19 surges.^3^

### Data collection and outcome measures

Data on AU and AMR spanning the years 2018–2021 was collected retrospectively. The primary outcome measure was AU changes before and after the COVID-19 pandemic, including APBL and non-APBL use. AU was measured as days of therapy per 1,000 patient days (DOT per 1,000 patient days). The prepandemic period was from January 1, 2018, to December 31, 2019, and the pandemic response period was from January 1, 2020, to December 31, 2021. Secondary outcomes included the incidence rate of extended-spectrum beta-lactamase (ESBL)-producing organisms and carbapenem-resistant Enterobacterales (CRE) during the study period.

## Results

### Antimicrobial use

The average quarterly prepandemic DOT per 1,000 patient days was 362, with 366.5 and 359 in 2018 and 2019 respectively. In the pandemic period, the average quarterly DOT per 1,000 patient days declined to 353, with an initial increase in 2020 to 391 and a subsequent decline in 2021 to 318. The average quarterly DOT per 1,000 patient days increased from 359 to 391 between 2019 and 2020, respectively, representing an 8.7% increase. However, in 2021, the average quarterly DOT per 1,000 patient days decreased to 318, representing an 18.6% decrease from the first pandemic year.

### Antimicrobial use and COVID-19 surges

**Figure 1.**
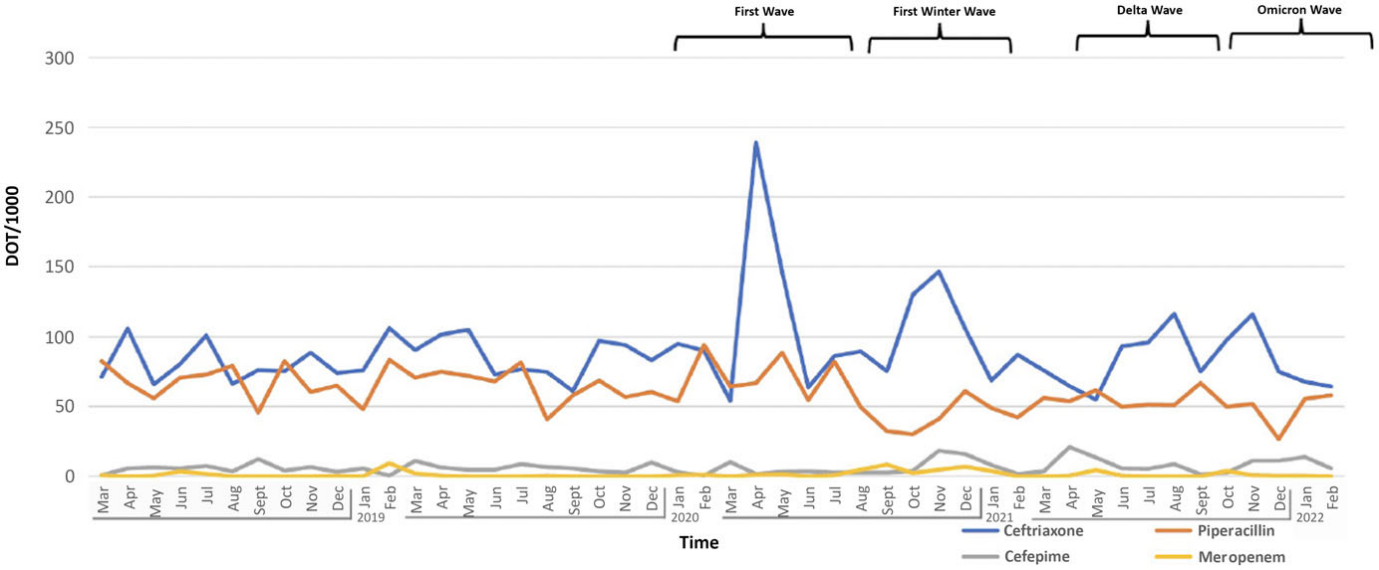
Monthly DOT per 1,000 patient days for ceftriaxone, cefepime, piperacillin, and meropenem during the study period.

Quarterly DOT per 1,000 patient days increased during COVID-19 surges, peaking at 440 DOT per 1,000 patient days during the initial COVID-19 surge, 22% above the prepandemic average. Smaller peaks occurred in each subsequent surge with 429 during the first winter, 351 during the delta surge, and 317 during the omicron surge. A 29% decrease in peak DOT per 1,000 patient days occurred from the first surge to the omicron surge. The average quarterly DOT per 1,000 patient days for ceftriaxone increased from 83 before the pandemic to 97 during the pandemic. Ceftriaxone DOT per 1,000 patient days increased during surges, reflecting our COVID-19 guidelines (Figure 1). Peaks declined each subsequent surge, from a peak of 239 in the first surge to 75 during omicron, representing a 68% decrease. The average monthly DOT per 1,000 patient days for APBL decreased from 73.51 to 63.21 (Supplement 2).

### Antimicrobial resistance

**Figure 2.**
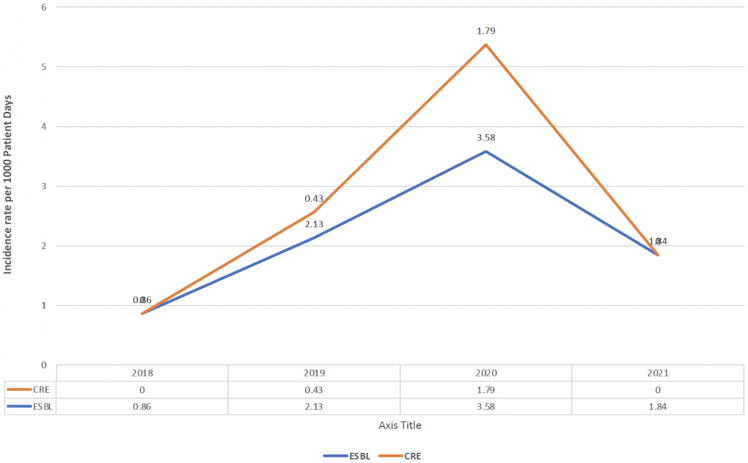
Incidence rate per 1,000 patient days for extended-spectrum beta-lactamase (ESBL)-producing organisms and carbapenem-resistant Enterobacterales (CRE).

The incidence rate per 1,000 patient days for ESBL-producing organisms increased from 0.86 in 2018 to 2.13 in 2019 and further to 3.58 in 2020, before decreasing to 1.84 in 2021. For CRE, the incidence increased from 0 in 2018 to .43 in 2019 and further to 1.79 in 2020, before returning to 0 in 2021 (Figure 2).

## Conclusion

Our study highlights the pivotal role of ASP in curbing AU at a time of high demand due to the COVID-19 pandemic. By incorporating COVID-19-specific elements into our ASP framework, we were able to guide AU during this global health crisis. Notably, while the overall AU increased during the initial year of the pandemic, our ASP interventions effectively steered away from the use of APBLs and led to a decline in the use of these agents across the pandemic. We observed a consistent reduction in AU peaks with each successive COVID-19 surge, indicating a growing adherence to ASP recommendations and comfort with treating viral LRTIs without antibacterial agents (Figure 1). During the COVID-19 pandemic, AMR increased nationwide.^5^ We observed an increase in AMR at our facility during the first year of the pandemic as well. However, a subsequent decline in AMR was seen as AU decreased. This suggests that ASP not only guided AU but also may have contributed to the mitigation of AMR.

ASP programs played a pivotal role during the COVID-19 pandemic, such as deploying novel therapeutics, disseminating treatment protocols, and implementing drug formulary restrictions.^8,9^ Leveraging existing ASP interventions and infrastructure may also be a crucial strategy for future pandemics. Our facility had an existing syndrome-based stewardship intervention targeting common infectious syndromes such as urinary tract infections, community-acquired pneumonia, and soft tissue infections that led to reductions in antipseudomonal beta-lactam use, AMR, *C. difficile* rates, and costs.^10^ By adapting this intervention to include COVID-19-specific elements, we successfully managed the challenges posed by the pandemic.

Limitations to this study include its single-center and retrospective design. However, our findings underscore the adaptability and effectiveness of ASP in optimizing AU during respiratory pandemics like COVID-19 and its effectiveness in a small community hospital. Tailored stewardship strategies can optimize AU and curb AMR. Lessons learned from this study may have implications for future pandemic preparedness and response.

## Supporting information

Mena Lora et al. supplementary material Mena Lora et al. supplementary material

## Supplementary material

For supplementary material accompanying this paper visit http://doi.org/10.1017/ash.2024.383. click here to view supplementary material
