# Supplementary material for: Levees for a hundred-year flood: impact of a syndrome-based antimicrobial stewardship intervention for coronavirus disease 2019 on antimicrobial use and resistance
Source: Antimicrob Steward Healthc Epidemiol. 2024 Sep 18;4(1):e131. doi: 10.1017/ash.2024.383 (PMC11427971; doi:10.1017/ash.2024.383)

**SUPPLEMENTS**

Supplement 1. Electronic medical record COVID-19 order set


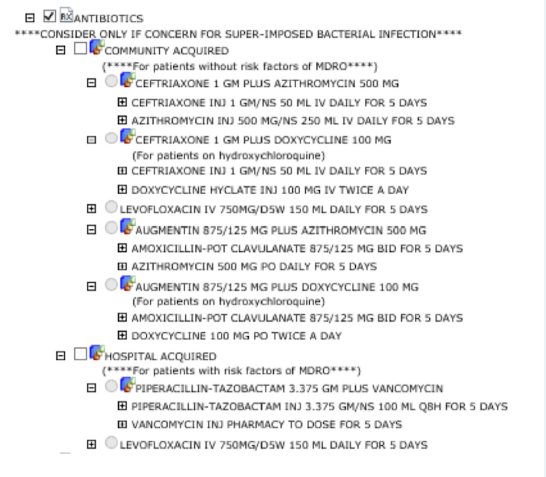


Supplement 2. DOT/1000 for anti-Pseudomonal beta-lactams and non-APBL before and after the intervention.


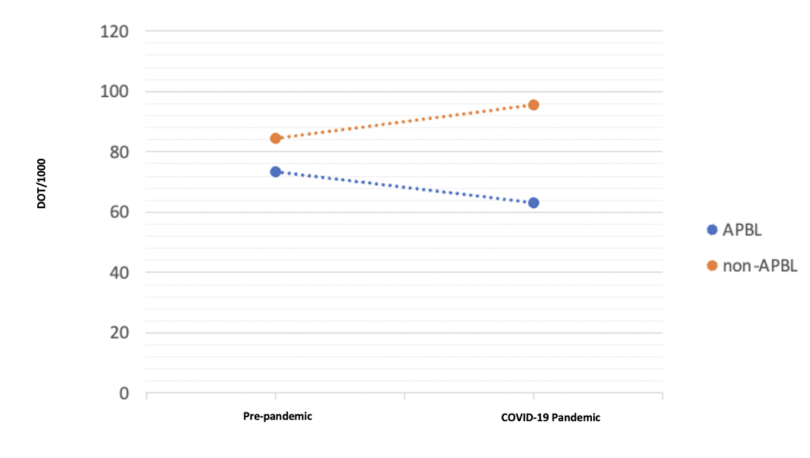

Supplement: Mena Lora et al. supplementary material [file S2732494X24003838sup001.docx]
